# The Role of Marrow Microenvironment in the Growth and Development of Malignant Plasma Cells in Multiple Myeloma

**DOI:** 10.3390/ijms22094462

**Published:** 2021-04-24

**Authors:** Nikolaos Giannakoulas, Ioannis Ntanasis-Stathopoulos, Evangelos Terpos

**Affiliations:** 1 Department of Hematology of University Hospital of Larisa, Faculty of Medicine, University of Thessaly, 41110 Larisa, Greece; ngiannak@med.uth.gr; 2 Department of Clinical Therapeutics, School of Medicine, National and Kapodistrian University of Athens, 11528 Athens, Greece; johnntanasis@med.uoa.gr

**Keywords:** multiple myeloma, plasma cells, bone marrow, microenvironment, cytokines, bone disease, mesenchymal stem cells, osteoblasts, osteoclasts, immune cells

## Abstract

The development and effectiveness of novel therapies in multiple myeloma have been established in large clinical trials. However, multiple myeloma remains an incurable malignancy despite significant therapeutic advances. Accumulating data have elucidated our understanding of the genetic background of the malignant plasma cells along with the role of the bone marrow microenvironment. Currently, the interaction among myeloma cells and the components of the microenvironment are considered crucial in multiple myeloma pathogenesis. Adhesion molecules, cytokines and the extracellular matrix play a critical role in the interplay among genetically transformed clonal plasma cells and stromal cells, leading to the proliferation, progression and survival of myeloma cells. In this review, we provide an overview of the multifaceted role of the bone marrow microenvironment in the growth and development of malignant plasma cells in multiple myeloma.

## 1. Introduction

Multiple myeloma (MM) is a heterogeneous hematologic malignancy of clonal plasma cells, which almost always produce a monoclonal immunoglobulin detected in the serum and/or in the urine. Plasma cells are hosted in the bone marrow and their proliferation is fully dependent on their interaction with almost all components of the bone marrow microenvironment (BMM) [1,2].

MM accounts for about 10% of hematologic malignancies and it is the second most frequent hematologic neoplasm after lymphomas. The median age of patients with MM is nearly 70 years old with an increasing incidence with age. Although a significant advancement in the understanding of pathophysiology and the availability of novel treatments has been achieved, MM remains an incurable disease [3].

The exact etiology of MM is still unknown. Genetic and environmental causes have a significant role in the development of MM. Genetic predisposition, antigenic stimulation, and exposure to radiation or chemicals are some of the possible agents that initiate the process of “myelomagenesis” [4]. Α premalignant phase of monoclonal gammopathy of unidentified significance (MGUS) almost always precedes the symptomatic MM phase [5,6]. In addition, the BMM plays a key role in that process through several interactions with myeloma plasma cells. Genetic, molecular and clinical heterogeneity is a hallmark characteristic of multiple myeloma patients [7].

The International Myeloma Working Group (IMWG) has updated the descriptions of MGUS, smoldering multiple myeloma (SMM) and symptomatic/active myeloma [8]. Thus, the definition of active myeloma includes not only the well-known CRAB criteria (anemia, renal insufficiency, hypercalcemia, lytic bone disease), but also at least one biomarker of malignancy such as the presence of more than 60% bone marrow clonal plasma cells, an abnormal kappa/lambda ratio of more than 100 and the manifestation of more than one bone lesion in a magnetic resonance imaging (MRI) or positron emission tomography/computed tomography (PET/CT) scan. The risk stratification of MM patients is based on clinical and laboratory characteristics as well as on cytogenetic abnormalities in plasma cells of MM patients. For newly diagnosed MM (NDMM) disease staging, the IMWG has suggested the revised international staging system (R-ISS), which is more accurate in terms of disease prognosis and is based on the combination of the International Staging System (ISS), the presence of certain chromosomal abnormalities identified by fluorescent in situ hybridization (FISH) in CD138+ selected plasma cells, and the value of serum lactate dehydrogenase (LDH) [8,9].

## 2. Pathogenesis of MM

Plasma cells (PCs) are irreversibly differentiated cells of the B cell compartment that remain in the bone marrow and produce serum immunoglobulins. Bone marrow myeloma cells have limited self-renewal and proliferative capacity in vivo.

The origin of the malignant plasma cells remains obscure. Although the plasma cell is the predominant cell in MM, there are data showing the existence of a precursor compartment since a number of peripheral blood lymphocytes have the unique paraprotein isotype and clonal immunoglobulin gene rearrangements identical to those seen in the bone marrow myeloma plasma cells. It is possible that myeloma cells are evolving from a memory B cell or plasmablast from the germinal centers of the lymph nodes [10,11,12,13]. The identification of the myeloma stem cell is still under investigation [14].

The development of MM is a multistep transformation process of the malignant cells within the BMM [15]. In almost all patients who develop multiple myeloma, a premalignant phase of MGUS can be identified [16]. The normal plasma cell is transformed to the premalignant MGUS/SMM state and eventually to symptomatic multiple myeloma through a number of sequential genetic and epigenetic events accumulated in the malignant plasma cell, several changes in the BMM which support the myeloma clone and probably a failure of the immune system to eliminate the malignant clone [16].

In MM patients, the karyotypic analysis usually shows several recurrent numerical abnormalities such as hyperdiploidy, loss of chromosome 13 and specific translocations involving the 14q32 locus [17,18,19]. The use of novel molecular technologies enables the identification of new subgroups and novel pathogenic pathways in MM, offering the ability of more accurately understanding the pathophysiology of the disease in addition to the elaboration of new targeted therapies. 

In 2006, a new grouping system based on microarray gene expression profiling data from the University of Arkansas for Medical Sciences (UAMS) classified seven subclasses of myeloma [20,21]. The first is defined by t(4;14) and multiple myeloma SET domain (MMSET) and/or fibroblast growth factor receptor 3 (FGFR3) genes overexpression. The second class is defined by t(14;16) or t(14;20), which leads to the upregulation of the MAF gene. The groups CD1 and CD2 are characterized by CCND1 or CCND3 upregulation due to t(11;14) or t(6;14). Hyperdiploidy is the main characteristic of the fifth group. The last two groups were described by low occurrence of bone disease due to low dickkopf 1 (DKK1) expression and high expression of genes involved in proliferation.

### Role of Oncogenes/Suppressor Genes in MM

In most malignancies, including MM, important cellular functions such as proliferation, growth and apoptosis are controlled by several oncogenes and suppressor genes that have been found to be deregulated in the context of malignancy. Beyond initial recurrent abnormalities such as hyperdiploidy, loss of chromosome 13 and non-random translocations involving the 14q32 locus, secondary genetic events such as deletions (13q, 17p), somatic mutations in RAS oncogenes and BRAF, the deregulation of bcl-2 and c-myc proteins, loss of tumor suppressor gene product function and other abnormalities highly contribute to MM progression [22]. In particular, retinoblastoma protein (pRB) seems to be constitutively phosphorylated in myeloma cells and interleukin 6 (IL-6) further shifts pRB to its phosphorylated form [23]. Cyclin D deregulation is a rather initial and uniting incident in MM pathogenesis [24]. CCND2 expression is regulated by post-transcriptional modifications [25]. Cyclin D1 is overexpressed in a high proportion of MM cells with t(11;14) (q13;q32) [26,27]. Interestingly, MM patients with CCND1 overexpression have poor prognosis [28,29]. The RAS/RAF/MEK/ERK pathway has a key role in multiple functions of plasma cells and angiogenesis. Mutations of KRAS/NRAS/BRAF are found in almost half of MM patients [30] and in an even higher percentage of patients with RRMM [31,32], resulting in more aggressive clinical behavior and poorer prognosis [32]. The translocation t(4;14) leads to amplified expression of FGFR3, which also activates the RAS/mitogen-activated protein kinase (MAPK) pathway [33]. The overexpression of BCL-2 makes myeloma cells resistant to apoptosis. Bcl-2 is probably a critical survival factor for myeloma cells in the hypo-proliferative early stages of the disease since it seems to lose its importance in the aggressive proliferative phase of the disease. High expression of cyclin D1 and BCL-2 and low BCL-XL and MCL-1 are detected in MM cell lines and primary patient samples with t(11;14). Venetoclax, a bcl-2 inhibitor, has shown encouraging results in these patients [34]. Mutations of p53 are uncommon in MM (10%) and are mainly observed in the terminal stage of the disease [35]. IL-6, which has a crucial role in MM pathogenesis, can directly trigger PI3K [36] and activate AKT [37,38] and the RAS/MAPK pathway [39]. In MM, histone deacetylase (HDAC) function is deregulated and may result in the upregulation of oncogenes [40]. Overall, the unique molecular characteristics in MM play a key role in the interactions between myeloma cells and the components of the BMM.

## 3. The BMM in MM

The BMM consists of several types of cells that provide all the necessary factors required for the development of hematopoietic stem cells (HSCs). In particular, it contains the cellular component (hematopoietic and non-hematopoietic or stromal cells, such as osteoblasts, osteoclasts, fibroblasts, adipocytes, myocytes, endothelial cells, lymphocytes, dendritic cells and macrophages), the extracellular matrix (several types of collagen, laminin, fibronectin, thrombospondin, proteoglycans and hemonectin) and the soluble part (cytokines, growth factors, soluble isoforms of cell adhesion molecules, e.g., serum vascular cell adhesion protein 1 (sVCAM-1), serum intercellular adhesion molecule 1 (sICAM-1), sP-selectin, sE-selectin). Normal hemopoiesis is maintained by the interaction of the above components [41]. 

The pathogenesis of most hematologic malignancies is multifactorial. In several hematological malignancies, the properties of the HCSs are determined by the BM milieu [42]. Although the initiation and progression of tumor cells is based on their genetic defects, the BMM, which is a dynamic niche, highly contributes to malignant transformation and disease progression via a variety of interactions with malignant cells. The MM microenvironment has been studied in vitro (e.g., 5TMM series) and in several murine models [43]. The results of such studies have limitations since there are differences between murine and human MM cells. A typical MM microenvironment contains several cell types and mediators, including mesenchymal cells, osteoblasts and osteoclasts, adipocytes and several immunomodulatory cells (macrophages, natural killer (NK) cells, regulatory T cells (Tregs), etc.). In MM, a number of biological phenomena are affected by tumor–host intramedullary interactions. The direct interaction of myeloma cells with stromal cells and other cellular components of the bone marrow, mostly via adhesion molecules, as well as indirect interaction via several cytokines result in the activation and proliferation of myeloma cells [44,45,46,47]. A number of cytokines such as IL-6, B-cell-activating factor (BAFF), a proliferation inducing ligand (APRIL), as well as other factors such as Receptor Activator of NF-κB (RANK), Vascular Endothelial Growth factor (VEGF), Colony stimulating factor-1 (CSF1), Fibroblast Growth factor-2 (FGF-2), angiopoietin-1, and IL-8 can boost the proliferation, survival, migration and drug resistance of MM cells [48,49]. MM cells may also naturally employ iNOS-cGMP signaling, c-Myc, Src and ERK kinases to proliferate [50,51,52]. Moreover, all these signal cascades lead to bone disease and angiogenesis. Along with activated inflammatory agents, a hypoxic microenvironment with reactive oxygen species (ROS) and reactive nitrogen intermediates (RNI) can further enhance genomic instability, select quiescent clones and also promote drug resistance [53,54]. The effects of hypoxia are still under investigation.

Human BMM cells are frequently insufficiently preserved in tissue biopsies for detailed studies. The sequencing of circulating tumor cells and cell-free DNA might aid in the better understanding of disease heterogeneity and evolution in multiple myeloma [55,56,57]. Intravital microscopy and other imaging techniques have been used for studying the relations between normal and malignant hematopoietic cells with their microenvironment [58]. Ex vivo models have also been developed to simulate the BMM and predict the response to treatment [59].

### 3.1. Important Cellular Components of the BMM in Myelomatogenesis

#### 3.1.1. Mesenchymal Stem Cells

Mesenchymal stem cells (MSCs) support, maintain and regulate the normal HSCs, since they have several functions such as self-renewal, differentiation, cell signaling, tumor homing and immunomodulation [50,60]. MSCs’ multiple functions are mediated via several molecules, such as adhesion molecules (VCAM-1, ICAM-1 and ALCAM), growth factors (stem cell factor (SCF), transforming growth factor β (TGF-β), epidermal growth factor (EGF), Granulocyte Macrophage Colony-Stimulating Factor (GM-CSF) and hepatocyte growth factor (HGF)), cytokines (interleukins IL-1α, IL-1β, IL-6, IL-7 and IL-8), angiogenic factors (VEGF and PDGF) and immunomodulatory molecules (PGE2, HLA-G and indoleamine 2,3-dioxygenase) [61,62,63,64,65,66,67]. 

However, in several hematological malignancies, MSCs are deregulated and contribute to the disease initiation and/or progression. MSCs from myeloma patients (MM-MSCs) interrelate with MM cells and change the expression of certain angiogenic and growth factors (such as CD40/40L, VCAM-1, ICAM-1, LFA-3 and HO-1) and several cytokines (IL-6, IL-10 TGFb1, macrophage inflammatory protein-1b (MIP-1b) and IL-7) [68,69,70,71,72,73]. Some abnormalities of the MM-MSCs decrease the osteoblastic activity or increase myeloma cells’ drug-resistance, like bortezomib resistance through increased NF-kB signaling due to the secretion of IL-8 and other factors [74].

MSCs produce soluble factors including extracellular vesicles (EVs) that, according to their characteristics, are divided into exosomes and micro-vesicles (MVs) [75]. The role of MM-MSC-EVs has been largely investigated. MSC-EVs from healthy individuals inhibit the MM cell proliferation in vitro, though their counterparts from MM patients have the opposite effect by activating the AKT pathway and inhibiting the p38, p53, and c-Jun N-terminal kinase (JNK) pathway [76,77,78,79]. MM-MSC-EVs express high levels of IL6, CCL2, and fibronectin and low levels of the tumor suppressor mir15a [75,76,78,79]. It has also been suggested that MM EVs block the differentiation and mineralization of BM-MSCs [80] and also participate in tumor cell homing in vivo. Increased phosphorylated AKT and ERK, cyclin D2, CDK4 and bcl-xl and low cleaved caspase 3 and poly ADP ribose polymerase (PARP) have been found in MM cells after co-culture with BMSCs. These pathways are thought to have high importance in the fate of MM cells [75,76,78].

#### 3.1.2. Osteoblasts, Osteoclasts and Osteocytes

Normal bone remodeling is based on the balance between bone formation by osteoblasts (OBs) and degradation by osteoclasts (OCs) [81]. Bone damage in MM is primarily caused by increased osteoclastogenesis, mediated by RANKL and MIP-1a [82,83,84,85]. Moreover, osteoprotegerin (OPG), a RANKL antagonist, and DKK1 are suppressed by MM cells [81]. Several anti-myeloma agents exert a beneficial effect on bone remodeling by restoring the homeostasis of the BMM [86,87,88,89].

In healthy conditions, OBs mineralize and rebuild healthy bone matrices after activation by factors released during OC activation [89,90]. In MM, OB growth and function is significantly reduced; so, the bone homeostasis is shifted in favor of osteoclastic bone resorption [91,92]. Decorin is a proteoglycan produced by OBs that directly inhibits the proliferation and survival of MM cells in vitro by inducing the apoptosis and arrest of the MM cell cycle [93]. The interaction between OBs and MM cells supports the growth of MM cells, provides a suitable niche to host dormant MM cells and suppresses OB differentiation via the production of soluble factors such as osteocalcin, osteopontin, FGF and TGFb [90].

OCs normally mediate bone resorption. In MM, OCs play a key role due to their increased and uncontrolled activation [94]. The lysis of bone tissue releases calcium, growth factors and extracellular matrix (ECM) proteins that promote tumor growth and survival [95,96,97,98]. The RANK-RANKL/OPG signaling pathway, which is the most important regulatory pathway that maintains bone remodeling, is highly deregulated in MM with augmented RANKL expression and reduced OPG [82]. RANKL, produced by OB-progenitor cells and MM cells, binds and neutralizes OPG, which normally inhibits RANK/RANK-L signaling and prevents OC activation. Importantly, RANKL-targeted therapeutics are being integrated in MM treatment with denosumab, an anti-RANKL monoclonal antibody, having a positive effect on myeloma bone disease [99]. OCs express Semaphorin 4D, which acts as a mediator of OC–OB interaction by binding to its receptor Plexin-B1, and eventually prevents bone formation by OBs [100]. OCs can activate quiescent myeloma cells by modifying the endosteal niche and promote angiogenesis via the production of pro-angiogenic agents [101].

Osteocytes that are embedded in the bone matrix arise from the differentiation of osteoblasts and make up 95% of bone cells [102,103]. Under physiological conditions, they regulate the reaction to small bone fractures by secreting in the BMM factors such as sclerostin and RANKL, modulating OB and OC activity. In MM, the number of osteocytes is decreased as they show increased apoptosis, and they play a key role in myeloma bone disease [101]. Sclerostin, which is a Wnt inhibitor, diminishes the OB differentiation [104]. In MM patients, the circulating sclerostin levels are correlated with bone disease severity [105]. Anti-sclerostin antibodies are currently under clinical investigation [106].

#### 3.1.3. Adipocytes

Bone marrow fat (BMF) has a significant role in various functions of myeloma cells in the BMM. Interestingly, BMF disappears during disease progression, suggesting that adipocytes’ role may be particularly important in the initial stages of the disease [107,108]. 

Adipocytes offer energy in the BM. They also produce adipokines (leptin and adiponectin) and growth factors (e.g., IL-6, tumor necrosis factor a (TNFa), monocyte chemoattractant protein 1 (MCP-1)), which play a role in myelomagenesis or disease progression. Adipocytes from MM patient femoral biopsies can maintain myeloma growth in vitro and may protect MM cells from chemotherapy-induced apoptosis [109]. 

Adipocytes arising from MSCs interact with MM cells in the BMM. BMF tissue is associated with an increased risk of MM occurrence as obesity increases the risk of MM development. High levels of adiponectin reduce the growth of MM cells and diminish angiogenesis through several signaling pathways (cyclic AMP-dependent protein kinase A, signal transducer and activator of transcription 3 (STAT3), MAPK, b-catenin and PI3K/AKT). On the contrary, the deficiency in adiponectin observed in obesity diminishes various signaling pathways that are involved in the prevention of proliferation, migration and drug resistance in MM cells [110,111]. In addition to the above, leptin, which is secreted only by BMSCs in the BMM, plays a role in the evolution from MGUS to MM by regulating the OPG/RANKL pathway [112,113]. Elevated serum levels of leptin have been found in MM patients at diagnosis compared to healthy individuals and decreased after treatment [112]. Resistin, which is another adipokine secreted by adipocytes, protects MM cells from chemotherapy-induced apoptosis [114].

Overall, the abovementioned components of the cellular compartment of the BMM present interrelated interactions with MM cells, which favor the hosting and the survival of MM cells in the bone marrow milieu.

### 3.2. Adhesion Molecules

Myeloma cells in the BMM interrelate with stromal cells (BMSCs), osteoblasts, osteoclasts, lymphocytes and endothelial cells. This interaction is mediated via cell adhesion molecules (CAMs) such as CD44 (H-CAM), CD56 (N-CAM), members of the CD49 integrin family, including very late antigen 4 (VLA-4) and 5 (VLA-5), lymphocyte function-associated antigen 1, syndecan-1 and selectin. All these molecules have a crucial role in myeloma pathogenesis. More specifically, VLA-4, which binds to VCAM-1 or fibronectin, and LFA (leucocyte function associated antigen)-1, which binds to ICAM-1, bring myeloma cells in contact with BMSCs, respectively [115,116,117,118]. This activates the p42/44 MAPK and NF-kB, resulting in the expression of more adhesion molecules on myeloma cells and BMSCs. Then, an increased production of cytokines, particularly IL-6 and VEGF, stimulates both plasma cells and angiogenesis. Myeloma cells exposed to IL-6 show increased STAT3 phosphorylation, which leads to the activation of genes involved in proliferation and survival [119].

Another interaction system involved in motility and cytoskeletal changes is that of CXC chemokine ligand (CXCL)-12 expressed by BMSCs and its receptor CXC chemokine receptor (CXCR)-4 on MM cells [120]. Furthermore, CXCL-12 upregulates VLA-4, enhancing the adhesion of myeloma cells to BMSCs and cytokine production [121]. VLA-5 has also been associated with the adherence of MM cells to the bone marrow [118]. The gain of CD11b and LFA-1 combined with the loss of CD56, VLA-5 and syndecan-I expression is linked with the migration of plasma cells from the bone marrow and the development of plasma cell leukemia [122]. Selectin, another adhesion molecule, modulates the contacts between MM cells and neighboring stromal cells [123]. Adhesion molecules may also have a role in treatment response [124].

Overall, adhesion molecules orchestrate the cell–cell interactions between MM cells and the cells of the BMM by bringing them in close proximity and transducing signals from one another. 

### 3.3. Growth Factors-Cytokines

The growth and persistence of malignant myeloma plasma cells depend on the existence of cytokines and growth factors, which are mostly produced by stromal cells and osteoblasts. IL-6, stromal cell-derived factor 1 (SDF1), MIP-1, IL-10, insulin growth factor 1 (IGF-1), FGF, VEGF, HGF, Wnt-family members and others are some of the growth factors and cytokines involved in MM development [44,125]. Herein, we discuss the most relevant cytokines for the BMM.

IL-6, which is secreted by several stromal cells after IL-1 and TNF stimulation, provokes the production of acute phase reaction proteins. IL-6 is also involved in the proliferation of normal plasmablastic cells and terminal differentiation of these plasmablasts to non-dividing plasma cells [126].

The role of IL-6 in the pathogenesis of MM is crucial [127,128,129]. In MM, IL-6 is the key proliferation factor for immature plasma cells but not a differentiation factor since myeloma cells lack terminal differentiation. IL-6 highly contributes to MM cell proliferation via phosphorylation of the pRb [23]. The IL-6 secretion is stimulated by the interaction between myeloma cells and BMSCs. Although IL-6 is also produced by myeloma cells and an autocrine loop is active in MM, it is considered that it is almost exclusively produced by the BMM following the adhesion of myeloma cells to stromal and bone cells and in much higher amounts than in normal individuals. Several in vitro studies have shown the implication of IL-6 in the progression of MM. Serum IL-6 levels increase from MGUS to active MM status and are associated with disease aggressiveness [130]. Although the soluble receptor for IL-6 (sIL-6R) is detected in the serum and urine of healthy controls, it is highly increased in MM patients but its significance remains uncertain [131,132,133,134,135]. IL-6 is also partly responsible for the abnormalities observed in the immune system. Dendritic cells (DCs) of MM patients show lower expression of HLA-DR, CD40 and CD80 antigens and reduced T cell activation due to compromised antigen presentation compared with normal individuals [136]. This functionally defective phenotype of dendritic cells is mediated by increased IL-6 [136]. Ultimately, IL-6, which is also secreted by osteoclasts, increases the number of MM cells and favors bone resorption via increased IL-17 production by T cells, which in turn upregulates RANKL and increases osteoclast formation [137,138,139,140].

Granulocyte colony-stimulating factor (GCSF) has several similarities to IL-6 [141,142]. In vivo, GCSF may be a strong growth factor for MM cells [129]. In addition, both GCSF and IL-6 stimulate angiogenesis and tumor growth by altering signaling pathways like STAT3 in neutrophils [143].

IL-10, produced by T regulatory cells or myeloma cells, also contributes to the development of MM. Higher IL-10 serum levels are detected in MM patients compared with normal individuals and are also correlated with lower response rates and poor prognosis [144]. IL-10 affects several functions of the immune system, such as macrophage activation, cytokine production and antigen presentation [145], increases plasma cell proliferation and angiogenesis [146] and possibly promotes the emergence of other malignancies [147].

Overall, the soluble signaling molecules are produced by both MM cells and cells of the BMM and sustain MM survival and proliferation in the bone marrow.

## 4. Angiogenesis in MM

Since its first description in 1999, an increased neovascularization of the bone marrow is considered a prominent characteristic of MM [148]. Several neo-angiogenic factors, produced from BMSCs (VEGF, bFGF and TGFb) and from plasma cells (VEGF, FGFb, IL-8 and TGFb) trigger the angiogenesis process [149,150]. These pro-angiogenic factors incite endothelial cells to develop new vessels. Vascular endothelial growth factor (VEGF), and especially VEGFa isoform, is the main pro-angiogenic molecule [151,152,153] that regulates normal as well as pathological angiogenesis. It contributes to increased neovascularization in MM. VEGF receptors are uncovered on the surface of stromal and myeloma cells. Thus, autocrine and paracrine feedback mechanisms are implicated [151,152]. The score of neovascularization in the bone marrow measured with anti CD34 and CD15 of the endothelium is augmented in patients with MM compared with normal individuals, and it also correlates with the disease status, e.g., increases during evolution from MGUS to SMM/MM [154,155,156,157].

VEGFR-2 receptor is largely expressed on plasma cells and endothelial cells in MM [158]. The stimulation of VEGFR-2 leads to the phosphorylation of MAP kinase and increased cell proliferation. The plasma cells also secrete VEGF-a, which activates through its receptor the angiopoietic cells and with an autocrine mechanism the neoplastic plasma cells activating the intracellular pathway RAF-1/MEK-1. The expression of VEGF and its receptor is induced in angiopoietic cells in order to avoid cell death in a hypoxic microenvironment. Proto-oncogenes such as RAS, Src and Fos are upregulated by VEGFa in MM patients [22]. The inhibition of VEGFR-2 and VEGF-a decreases myeloma cell growth [159]. Semaphorin, an anti-angiogenic factor that normally regulates VEGF activity, has been found in small quantities in MM patients [160,161]. 

Angiopoietins (Ang) 1 and 2 that are ligands for the vascular-specific tyrosine kinase TIE-2 receptor of the endothelial cells also have a significant role in angiogenesis in MM [162,163,164].

The Platelet-derived growth factor (PDGF-b) increases the expression of the protein c-myc, which in turn reduces the sensibility of myeloma cells to melphalan treatment. In addition, melphalan-resistant patients show c-myc over-expression and PDGF-b higher serum levels [165].

FGFb triggers neovascularization in MM bone marrow [166]. The main source of FGFb is the myeloma cells and its levels correlate with disease severity. FGFb stimulates stromal cells to secrete IL-6. Additionally, MM cell lines stimulated with IL-6 secrete higher amounts of FGFb [166].

Matrix metallopeptidases (MMP-1, MMP-2, MMP-9) are highly expressed in both myeloma cells and cells of the microenvironment [167,168,169]. They are probably mediators for VEGF and PDGF-b dependent mechanisms. The mechanisms that lead to the upregulation of MMP activity remain to be clarified.

Overall, increased angiogenesis is a hallmark of the BMM in MM, which may serve as a basis for the development of anti-angiogenetic therapeutics.

## 5. Immune Deregulation in BMM

The BMM contains several types of immune cells co-operating in order to facilitate normal hemopoiesis [170]. Osteomacs, which are tissue-specific macrophages, contribute to the regulation of HSCs in the BM niche. They guide HSCs’ homing, migration and activity status. Osteomacs may mediate the inflammatory response and serve as a reservoir of osteoclast precursors in the bone marrow milieu [171].

Immunological parameters such as T cells, NK cells and others have been found to have important alterations in adaptive and innate immunity between healthy individuals, MGUS and multiple myeloma patients. Oligoclonal CD8+ T cell expansion has been found in both MGUS and MM [172]. MM NK cells produce lower IFNγ and show functionally faulty features compared to NK cells in MGUS [173]. Macrophages enhance the proliferation and survival of MM cell lines and protect plasma cells from apoptosis via the production of IL-6 and other factors [50]. The function of immune cells in MM patients is also impaired [174]. MM patients are susceptible to infections and secondary malignancies. The numbers of B, NK and T cells in MM patients are lower than normal individuals [175,176]. The low levels of normal immunoglobulins are a typical characteristic of active MM, as well [3]. 

Compared to normal plasma cells, malignant plasma cells have increased HLA-1 and co-stimulatory molecules on their surface. There are contradictory results concerning the amount of HLA-1 expression on plasma cells as malignant plasma cells from MGUS progress to multiple myeloma [177].

DCs are dysfunctional in MM [136]. The direct interface of DCs and PCs resulted in PC persistence. In a recent in vitro model, the interaction between DCs-MM and T lymphocytes led DCs to secrete IL-6 and the immunosuppressive enzyme indoleamine 2,3 dioxygenase (IDO), resulting in the anergy of activated T cells and the differentiation of T cells into suppressive CD25 high/FOXP3+/CD4+ Treg [178]. 

Tregs in the bone marrow maintain the BMM homeostasis that can be despoiled by several conditions including the presence of cancer cells. The Th1/Th2 ratio is distorted in MM with reduced production of Th1 cytokines (IL-2 and IFNγ) and increased production of Th2 cytokines (IL-10 and IL-4). Tregs from MM patients fail to control T-cell expansion and function. Tregs cells also secrete IL-10 and TGF-β, stimulating MM persistence [179,180]. Furthermore, the increased CD200 expression by malignant plasma cells has been positively associated with an increased percentage of immunosuppressive Tregs and worse patient survival [181,182,183].

Th17 cells are increased in MM patients due to increased pro-inflammatory cytokines [138,139,180,184]. Th17 cells produce IL-17 and IL-22 and they are strongly related to the effect of IL-21, IL-22, IL-23 and IL-27, whose levels are increased in MM. All these increased cytokines suppress the normal immune responses and support the growth of plasma cells by protecting them from cytotoxic lymphocytes. Furthermore, IL-17 has an important role in the progress of bone disease as it determines an upregulation of RANKL on stromal cells. The amount of IL-17 in BM is correlated with high-risk clinical features [140]. 

Myeloid-derived suppressor cells (MDSCs) are partially responsible for the tumor escape from immune surveillance [185,186,187]. They exert their immunosuppressive action mostly on T lymphocytes. MDSCs are also involved in angiogenesis because they produce MMP-9 and in osteoclastogenesis because they act as osteoclast progenitors [81,185,186,187]. The significance of MDSCs in MM growth is under investigation. 

Molecules such as programmed death-ligand 1 (PD-L1) that inhibit antitumor immune response are extensively expressed on a variety of solid tumors and are the major mechanism of escaping immune surveillance. On the other hand, the cell surface PD-1 is expressed on several immune cells (T, B and NK cells) [188]. PD-L1 is over-expressed in myeloma cell lines and patients’ myeloma cells, while its ligand PD1 is detected on T cells in MM patients [189]. High expression of PDL1 has been observed in relapsed/refractory MM and correlated with poor prognosis [189]. Although anti-PDL1 antibodies enhance the cytotoxicity of NK cells against MM cells in vitro, clinical studies have not shown the anticipated benefit [190,191,192].

Overall, immune deregulation is another hallmark of the bone marrow milieu in MM that drives myeloma growth and proliferation.

## 6. Conclusions

**Figure 1:**
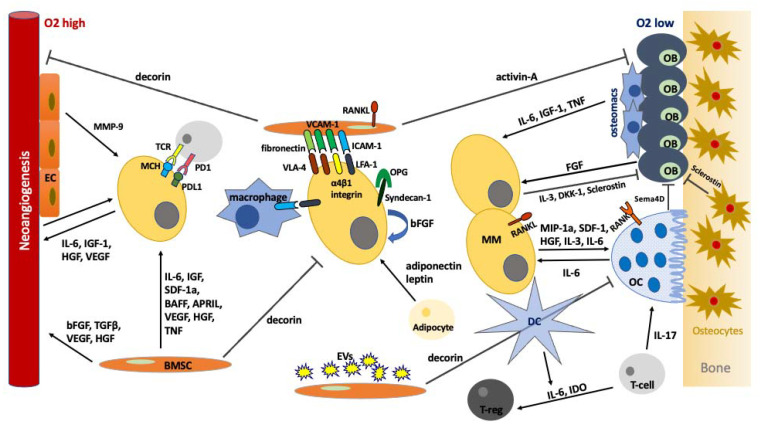
The complex interactions among the cellular components of the BMM in MM.

Multiple myeloma is a heterogeneous incurable hematologic malignancy. Multiple genetic alterations in a possibly genetically predisposed individual may initiate the myelomatogenesis process. Several types of cells in the microenvironment such as osteoblasts, osteoclasts, fibroblasts, adipocytes, myocytes, endothelial cells, lymphocytes, dendritic cells, macrophages, the extracellular matrix (several types of collagen, laminin, fibronectin, thrombospondin, proteoglycans and hemonectin), soluble factors such as cytokines, growth factors, and adhesion molecules contribute in a complex way to the development and progression of malignant plasma cells (Figure 1). The involved mechanisms are complex and rather not fully comprehended. Our deeper understanding of the key role of the BMM in MM pathogenesis may lead to the design and clinical application of novel targeted therapies (Table 1).

## Figures and Tables

**Table 1:** Current and investigational treatment approaches targeting BMM in MM.

Mechanism of Action	Drugs
Currently approved drugs	
Anti-CD38 mAb	Daratumumab, Isatuximab
Anti-SLAMF7 mAb	Elotuzumab
Anti-RANKL mAb	Denosumab
Anti-BCMA conjugated mAb	Belantamab mafodotin
Immunomodulators	Thalidomide, Lenalidomide, Pomalidomide
Histone deacetylase inhibitor	Panobinostat
Investigational drugs	
Immunomodulator	Iberdomide
Histone deacetylase inhibitors	Vorinostat, Abexinostat, Belinostat, Givinostat, Romidepsin, Entinostat, Tacedinaline
Activin inhibitor	Sotatercept
Hypoxia-activated prodrug	Evofosfamide
CXCR4 antagonist	Plerixafor
Anti-CXCR4 mAb	Ulocuplumab
Anti-PD-1 mAbs	Nivolumab, Pembrolizumab, Cemiplimab, Cetrelimab
Anti-PD-L1 mAbs	Atezolizumab, Avelumab, Pidilizumab
Anti-CTLA-4 mAb	Ipilimumab
Anti-CD52 mAb	Alemtuzumab
Anti-IL-6 mAb	Siltuximab
Anti-ICAM-1 mAb	BI-505
Anti-CD25 mAb	Daclizumab
Anti-IGF1R mAb	AVE1642
Anti-DKK1 mAb	BHQ880
Anti-VEGF mAb	Bevacizumab
Anti-BAFF mAb	Tabalumab
Bispecific T-cell engagers	Blinatumomab, AMG 701, REGN5458
Chimeric antigen receptor (CAR) T cells	Anti-CD19, anti-CD138, anti-BCMA, anti-SLAM7 CAR—T cells
BCL-2 inhibitor	Venetoclax
NF-κB inhibitor	DANFIN
RAS/RAF/MEK/ERK inhibitors	Sorafenib, Vemurafenib, Cobimetinib, Selumetinib
CDK4/6 inhibitor	Palbociclib
FGFR inhibitors	Dovitinib, BGJ398, MFGR1877S, AZD4547
PI3K/AKT/mTOR inhibitors	Clioquinol, SC-06, BEZ235, BAY80-6946, MK-2206

mAb: monoclonal antibody.

## Data Availability

The corresponding author will provide the data or will cooperate fully in obtaining and providing the data on which the manuscript is based for examination by the editors or their assignees.
